# Calidad de los registros del proceso en el área quirúrgica y posibles consecuencias para la seguridad del paciente

**DOI:** 10.23938/ASSN.1120

**Published:** 2025-09-22

**Authors:** Sixtina Perarnau-Pauner, Carmen Gomar-Sancho, Tània Estapé-Madinabeitia, Marina Mateu-Capell

**Affiliations:** 1 Fundación Hospital Sant Joan de Déu de Martorell Calidad Asistencial Martorell España; 2 Universidad de Vic. Universidad Central de Cataluña Facultad de Ciencias de la Salud Campus Manresa. Manresa Barcelona España; 3 Universidad de Vic. Universidad Central de Cataluña Facultad de Ciencias de la Salud Cátedra de Simulación y Seguridad del Paciente Campus Manresa. Manresa Barcelona España; 4 Instituto de Investigación e Innovación en Ciencias de la Vida y de la Salud en la Cataluña Central (IRIS-CC) Grupo de investigación Innovación Transformativa y Simulación (GRITS) Vic Barcelona España

**Keywords:** Registros clínicos, Omisiones de registro, Cirugía mayor, Seguridad del Paciente, Mejora de la calidad, Clinical records, Underregistration, Major Surgery, Patient Safety, Quality Improvement

## Abstract

**Fundamento:**

El objetivo es detectar defectos de cumplimentación (omisión/ilegibilidad) de los registros quirúrgicos, valorar su gravedad y potenciales consecuencias, con el fin de diseñar estrategias de mejora.

**Método:**

Se revisaron los registros clínicos de enfermería y anestesia (soporte papel), y cirugía (electrónico) de las intervenciones de cirugía mayor realizadas durante tres meses en el hospital de Martorell (España). Se identificaron defectos (omisiones e ilegibilidad), seleccionándose variables que presentaban defectos de cumplimentación en más del 50% de los registros o con potencial impacto en la seguridad del paciente. Un panel de expertos valoró mediante un cuestionario su gravedad (alta=70-89%, muy alta= >90%) y posibles consecuencias (pre-, intra-, postquirúrgicas y administrativas) y propuso mejoras.

**Resultados:**

Se analizaron las historias clínicas de 491 pacientes. La ilegibilidad fue casi inexistente, excepto para cuatro variables (≤10%). El grado de cumplimentación fue del 98%. Hubo 43 variables con defectos en >50% de registros o un impacto potencial y que se incluyeron en el cuestionario enviado a 29 expertos. La fiabilidad de sus opiniones fue muy alta (α=0,995; coeficientes de correlación intraclase individual=0,880 y promedio= 0,995). Las omisiones de prácticamente todas las variables se consideraron de gravedad alta/muy alta, con más consecuencias postoperatorias que intraoperatorias. La formación presencial de los equipos y la adecuación de los registros fueron las medidas de mejora más recomendadas.

**Conclusiones:**

Los registros quirúrgicos presentan omisiones graves/muy graves de cumplimentación con potenciales consecuencias post-operatorias sobre la seguridad del paciente. La formación mediante simulación se consideró la herramienta más eficaz para mejorarla.

## Abstract (en)

**Background:**

The objective is to detect defects in the completion of surgical records (omissions/illegibility), assess their severity and potential consequences, and design improvement strategies.

**Methods:**

The clinical records of nursing and anaesthesia (paper-based) and surgical records (electronic) for major surgical procedures performed over a three-month period at Martorell Hospital (Spain) were reviewed. Deficiencies (omissions and illegibility) were identified, and variables with completion deficiencies in more than 50% of the records or with potential impact on patient safety were selected. A panel of experts used a questionnaire to assess severity (high = 70-89%, very high = >90%) and possible consequences (pre-, intra-, post-surgical, and administrative), and proposed for improvement measures.

**Results:**

Medical records from 491 patients were analysed. Illegibility was almost non-existent, except for four variables (≤10%). The overall completion rate was 98%. Forty-three variables with defects in >50% of records or with potential impact were included in the questionnaire, which was sent to 29 experts. The reliability of their responses was very high (α=0.995; intraclass correlation coefficients: individual=0.880 and average=0.995). Omissions of nearly all variables were considered of high or very high severity, with postoperative consequences outweighing intraoperative ones. Face-to-face team training and record adaptation were the most frequently recommended improvement strategies.

**Conclusions:**

Surgical records show serious to very serious omissions with potential postoperative consequences for patient safety. Simulation-based training was considered the most effective tool for improvement.

## INTRODUCCIÓN

La comunicación ineficaz está implicada en el 80% de los errores médicos graves^1^ y, además de causar daños al paciente, provoca retrasos o errores en el tratamiento disminuyendo la seguridad del paciente (SdP). Los efectos adversos por fallos en la comunicación se producen en la transferencia de pacientes entre profesionales y disminuyen un 50% aplicando estrategias adecuadas^2^.

El bloque quirúrgico es un área de alta complejidad asistencial y organizativa, con múltiples acciones sobre el paciente en un período de tiempo corto. Durante el proceso quirúrgico intervienen distintos profesionales que se comunican información esencial, y en esa transferencia pueden surgir dificultades, ya sea en el quirófano, en las unidades de reanimación o en las plantas de hospitalización. Para los cuidados postoperatorios, por ejemplo, es esencial disponer de la información sobre el procedimiento quirúrgico disponible en el informe quirúrgico del cirujano. La transferencia inadecuada de información en procedimientos quirúrgicos exitosos puede causar complicaciones postoperatorias que hubieran sido evitables^3^^,^^4^. En nuestro conocimiento no se han descrito los problemas específicos de transferencia de información en el área quirúrgica ni su incidencia, aunque se ha publicado un aumento del riesgo de resultados adversos postoperatorios debidos a la ausencia de transferencia verbal entre relevos del personal de anestesia^5^. La transferencia verbal es muy frecuente en el área quirúrgica^3^^,^^4^ debido al ritmo de trabajo; aunque es útil para los interlocutores, no permite que dicha información esté disponible para el resto de los intervinientes en el proceso, ni permite cumplir que sea permanente mediante los registros^6^.

Los registros clínicos son herramientas de comunicación y transferencia de información fundamental entre profesionales que proporcionan evidencia de las decisiones clínicas y cuidados aplicados, indispensables desde el punto de vista clínico y médico-legal^7^^,^^8^. La calidad los registros mejora los procesos, los resultados y la adherencia a las evidencias científicas, siendo esenciales para la investigación^9^.

La lista de verificación de seguridad quirúrgica (LVSQ) de la OMS es un registro de uso extendido en el área quirúrgica que, aunque muestra problemas frecuentes de cumplimentación^10^, es eficaz para la SdP. Pero se trata de un *checklist* de la fase intraquirófano que permite compartir información solo entre las personas del equipo que se encuentra dentro del quirófano y que no abarca todas las transferencias de información que suceden en el circuito del área quirúrgica. No se dispone de información sobre un estándar de registro diseñado para garantizar la transferencia de información en las transferencias a través de los dispositivos por los que pasa el paciente dentro del área quirúrgica.

Los datos ausentes, incompletos o erróneos en los registros pueden tener consecuencias sobre la SdP al dificultar la toma de las decisiones adecuadas en las distintas fases asistenciales^4^^,^^9^ y son un área de mejora de la calidad asistencial^11^^,^^12^. Requieren evaluar su calidad y concienciar a los profesionales de su importancia. Es necesario detectar los puntos concretos de errores frecuentes y/o graves de los registros para priorizar las fases de un plan de mejora especifico^13^. Hasta donde sabemos no se ha estudiado la calidad los registros del área quirúrgica.

El Ministerio de Sanidad publicó un documento sobre práctica clínica segura y de calidad^13^, y el Servicio Catán de Salud publicó guías para algunos tipos de cirugía^14^. Aunque estos documentos abordan elementos de seguridad quirúrgica, no incluyen las consecuencias de un registro deficiente.

Por ello, indagamos la opinión de expertos sobre la gravedad y consecuencias de los registros incompletos, con el fin de establecer medidas de mejora para la calidad asistencial y de SdP quirúrgico de nuestro centro.

El objetivo de este estudio es revisar los registros quirúrgicos de cirugía mayor de nuestro centro identificando los fallos en la cumplimentación, por omisión o ilegibilidad, y obtener la valoración por los expertos sobre su potencial riesgo y consecuencias, así como las propuestas para poder fundamentar una estrategia de mejora de la calidad de los registros para mejorar la calidad asistencial y la SdP en el bloque quirúrgico de nuestro centro.

## METODOLOGÍA

Estudio transversal realizado en el Hospital Sant Joan de Déu de Martorell (Barcelona, España). El hospital pertenece a la red hospitalaria de utilización pública de Cataluña (SISCAT). Incluye las especialidades quirúrgicas de traumatología, ginecología, cirugía general, urología, cirugía vascular y dos unidades multidisciplinarias de cirugía. Se realizan anualmente 2.500 intervenciones de cirugía mayor en promedio en las que actúan sucesivamente 394 profesionales. El equipo quirúrgico lo constituyen el cirujano, un anestesiólogo, dos enfermeras quirúrgicas, una enfermera de apoyo a la anestesia, que recibe al paciente, y la enfermera de la unidad de recuperación postoperatoria (URPA).

Se incluyeron, por conveniencia, las intervenciones realizadas entre enero y marzo de 2020, de forma consecutiva. Los criterios de inclusión fueron: intervenciones de cirugía mayor (que representa alrededor del 25% anual de esta actividad), electivas y urgentes, de más de 24h de ingreso.

El estudio tuvo una etapa retrospectiva y otra prospectiva.

### Etapa retrospectiva

Se revisaron los registros cumplimentados por el personal de cirugía, anestesia y enfermería durante las intervenciones, así como los registros administrativos relacionados con el proceso quirúrgico. Todos ellos tenían campos prefijados y de texto libre. Mientras el informe del cirujano se cumplimenta solo en formato electrónico, el resto se registraron en soporte papel y fueron digitalizados en la historia clínica electrónica, proceso habitual de la institución. Se localizaron las historias clínicas de las personas intervenidas mediante un programa de registro institucional (Proh®) que contenía la información clínica y administrativa. Se revisaron los siguientes documentos:

- *Registro preoperatorio de enfermería*: en formato papel, con campos relacionados con datos clínicos del paciente, preparación quirúrgica, monitorización del paciente y actividades de enfermería, y campos de texto libre para los fármacos administrados.
- *Registro intraoperatorio de anestesia*: en formato papel, con campos para datos preanestésicos (valorados en consulta externa: peso, altura, exploración detallada de vía aérea, alergias) e intraanestésicos (constantes vitales, monitorización aplicada, parámetros de ventilación, medicación anticoagulante, intervención quirúrgica, inducción anestésica, tipo de anestesia, instrumentación de la vía aérea, balance hídrico, duración (horas), anestesistas), así como texto libre para consignar la administración de otros fármacos como relajantes o analgésicos (nombre del fármaco, vía, dosis y hora de administración) y observaciones anestésicas e incidencias.
- *Registro postquirúrgico de enfermería* realizado en la sala de reanimación post anestésica: en formato papel, contenía gráfica de registro de constantes vitales, valoración de dolor, datos relacionados con el alta (escalas de Aldrete y Bromage) e identificación de los profesionales de enfermería y anestesia; en texto libre se cumplimentan observaciones, transferencia a la unidad de hospitalización y medicación administrada (nombre del fármaco, vía, dosis y hora de administración).
- *Informe quirúrgico*: en formato electrónico, incluía datos identificativos de la persona sometida a cirugía, codificación del diagnóstico y del procedimiento, y facultativo responsable; la descripción del procedimiento quirúrgico se realizaba en un campo libre de escritura.

Se analizó la calidad de los registros clínicos cumplimentados por profesionales del área quirúrgica de nuestro centro. Se tomaron como referencia diferentes documentos publicados sobre práctica clínica segura y de calidad del Ministerio de Sanidad, y guías para algunos tipos de cirugía^13,14^. La valoración de los registros fue la siguiente:

- Se consideró *defecto* del registro cuando la variable que debía cumplimentarse se había omitido o, en el caso del soporte papel, era ilegible (ilegibilidad determinada por un documentalista);
- Cuando un dato no estaba registrado por no ser obligatorio, se definió como *no aplica*.

No se incluyó la información de la evaluación preanestésica realizada en consulta externa ni en la LVSQ, ya que se somete a una auditoria especifica en cada centro. En ningún caso se analizó la praxis asistencial ni se identificaron profesionales.

### Etapa prospectiva

En esta etapa se seleccionaron las variables que presentaban defectos/fallos en su cumplimentación en más del 50% en los registros, o que tenían un potencial impacto en la SdP en opinión de las personas investigadoras del estudio.

Se creó un cuestionario que incluía las variables seleccionadas. En él se solicitaba respuesta a dos preguntas en relación a cada variable, y elección de una o varias propuestas de mejora basadas en la literatura sobre formación de profesionales en temas de mejora de la calidad asistencial^15^^,^^16^ y en la experiencia de la institución de las personas autoras (Tabla 1). El cuestionario fue revisado por dos profesionales de anestesia, dos de cirugía y dos de enfermería quirúrgica, quienes realizaron aportaciones para su mejora. El cuestionario final fue elaborado en el programa REDCap®, generando un código QR de acceso que se envió por correo electrónico a las personas seleccionadas.

Mediante un muestreo en bola de nieve se contactó con personas con conocimientos y experiencia en el bloque quirúrgico o en SdP, con perfil de personal en activo de cirugía, anestesia, enfermería de procesos quirúrgicos y administrativo de bloque quirúrgico.

El período de recogida duró tres meses, con recordatorios.

**Tabla 1:** Contenido del cuestionario enviado a personas con experiencia en seguridad del paciente

¿Qué nivel de gravedad considera usted que tendría si esta variable no se encuentra marcada/anotada? Elegir una:	
☐	1= <50% (menos grave),
☐	2= 50-69%,
☐	3= 70-79%,
☐	4= 80-89%,
☐	5= 90-99%,
☐	6= 100% (extremadamente grave),
☐	no tengo conocimiento para responder a esta pregunta
¿En qué fase del proceso quirúrgico podrían tener consecuencias /complicaciones, si esta variable no está marcada/anotada? Elegir una o varias:	
☐	complicaciones clínicas prequirúrgicas
☐	complicaciones clínicas intraquirúrgicas
☐	complicaciones clínicas postquirúrgicas
☐	complicaciones administrativas
☐	otra complicación (redacción abierta)
☐	ninguna complicación
Propuestas de mejora Elegir una o varias:	
☐	formaciones telemáticas
☐	simulación clínica o aula invertida
☐	alertas informáticas de omisión
☐	autorías periódicas de los registros

Este estudio cumple con las normas y legislación vigente en la confidencialidad de datos. Fue aprobado por el Comité de Ética e Investigación Clínica del hospital Sant Joan de Déu de Martorell.

### Análisis estadístico

Las variables cuantitativas se describieron mediante media, desviación estándar (DE) y rango, y las categóricas frecuencias absolutas y relativas. Las respuestas a la pregunta del cuestionario sobre gravedad se agruparon en tres categorías: 1-2: <70% - omisión menos grave, 3-4: 70-89% - omisión grave y 5-6: ≥90% - omisión muy grave.

Se analizó la fiabilidad de la evaluación de los expertos mediante alfa de Cronbach y el grado de acuerdo mediante el coeficiente de correlación intraclase con su intervalo de confianza del 95% (IC95%). Los datos se recogieron en Excel (Microsoft) y se analizaron mediante el paquete estadístico SPSS-PC versión 27.0 (IBM).

## RESULTADOS

Se revisaron 546 historias clínicas. De ellas, 55 (10,07%) no correspondían a cirugía mayor, aunque así constara debido a error administrativo, por lo que se analizaron 491 historias clínicas.

Los problemas de legibilidad fueron casi inexistentes (<1%) excepto para una variable de los registros prequirúrgicos de enfermería (alergias del paciente, 10% de ilegibilidad) y para tres variables de los registros de anestesia, dos prequirúrgicas (peso del paciente y vía aérea Mallampati, ambas 5,5%) y una intraquirúrgica (incidencias anestésicas, 4%) (Tabla 2).

El grado de cumplimentación de las historias clínicas analizadas fue del 98%.

En los registros de enfermería, solo el 50% de las variables de las prequirúrgicas (5 de 10) y el 29,4% de las postquirúrgicas (5 de 17) tenían frecuencias de omisión <30%. Destaca la alta omisión de la frecuencia respiratoria, temperatura y glicemia y puntuación EVA, tanto en los registros pre-como postquirúrgicos. (Tabla 2).

En los registros de anestesia destacan las frecuentes omisiones de variables relacionadas con la vía aérea, el tipo de anestesia y de la ocurrencia o no de incidencias. Los resultados en relación con los fármacos muestran omisiones respecto del total de registros que debían contenerlos según la cirugía (Tabla 2).

En 20 intervenciones (4,07%) no se había realizado informe quirúrgico, por lo que se analizaron 471 informes. Se observó una alta frecuencia de omisión de incidencias (información postquirúrgica). No pudo valorarse el intervalo de tiempo entre el final de la intervención y su cumplimentación (Tabla 2).

**Tabla 2:** Porcentajes de omisiones e ilegibilidad de cada variable de los registros de enfermería, anestesia y cirugía, según el área

	Omitido		Ilegible	
	%		%	
*Registros de enfermería (n=491)*				
Variable	PreQ	PostQ	PreQ	PostQ
Alergias del paciente	4,00	9,8	10	0
Tensión arterial	17,11	9,8	0,2	0
Frecuencia cardíaca	19,76	29,1	0,2	0
Frecuencia respiratoria	93,69	90	0	0
Temperatura	78	78	0	0
Glicemia	82,69	40,3	0	0
Escala EVA de dolor	90,22	76,8	0	0
Sonda nasogástrica	43,38	81,7	0	0
Profilaxis antibiótica	29,9	96,5	0	0
Nombre analgésico no opioide	-	5,1	-	0
Nombre analgésico opioide	-	57,2	-	0
Nombre fluidoterapia	9,17	3,1	0	0
Test Aldrete	-	38,3	-	0
Alta de anestesia firmada	-	79,6	-	0
Alta de Enfermería	-	83,9	-	0
Destino a la salida del paciente	-	90,6	-	0
Llamada a la enfermera de hospitalización	-	64,2	-	0,20%
*Registros anestésicos (n=491)*				
Variable	PreQ	IntraQ	PreQ	IntraQ
Alergias del paciente	8,33	7,74	0,04	0,41
Tensión arterial	-	5,5	-	0
Frecuencia cardíaca	-	7,74	-	0,41
Vía aérea Mallampati	34,01	-	5,5	-
Historia intubación difícil	63,95	-	0,41	-
Ventilación facial difícil	40,73	-	0	-
Distancia tiromentoniana	52,55	-	0	-
Movilidad cervical	52,75	-	0	-
Diuresis	46,63	-	0	-
Peso del paciente	34,01	-	5,5	-
Altura del paciente	63,95	-	0,41	-
Sonda nasogástrica	-	96,74	-	0
Fármacos anestésicos	-	12,21	-	-
Fármaco antitrombótico	15,27	-	-	-
Analgésico no opioide	-	62,11	-	0
Analgésico opioides	-	11,2	-	0
Tipo de anestesia	-	69,2	-	0
Incidencias anestésicas	-	59,06	-	4,07
Incidencias hemodinámicas	-	68,8	-	0
Incidencias respiratorias	-	100	-	0
Incidencias neurológicas	-	95,93	-	0,2
Incidencias varias	-	98,78	-	0,2
*Informe quirúrgico*				
Variable	n	IntraQ		
Tipo de anestesia	471	94,5	-	-
Tipo de incisión	471	6,92	-	-
Cierre de la incisión	471	1,63	-	-
Recuento de gasas	471	78,28	-	-
Incidencias durante la cirugía	471	85,13	-	-
Profilaxis antibiótica	471	91,24	-	-
Recogida muestras para anatomía patológica	72	40,28	-	-
Recogida de muestras para biopsia	192	23,44	-	-
Recogida de muestras para cultivo	14	7,14	-	-

PreQ: área prequirúrgica; PostQ: postquirúrgica; IntraQ: intraquirúrgica; n: número de historias clínicas donde era indicado registrar esta variable; -: no aplica; EVA: escala visual analógica.

Hubo 43 variables que presentaban defectos en más del 50% en los registros o un potencial impacto en la seguridad del paciente y que se incluyeron en el cuestionario enviado a expertos.

De las 77 personas expertas contactadas, 29 respondieron al cuestionario (38,6%), la mayoría mujeres (n=23; 79,3%), con media de edad 50 años (DE=8,2; rango 37-72). Respecto a las categorías profesionales, diez eran profesionales de enfermería quirúrgica, cinco de anestesia y cinco de cirugía de diferentes especialidades, uno de enfermería y dos de medicina de hospitalización de cirugía, dos expertas en SdP y calidad asistencial y dos personales administrativos de bloque quirúrgico. Catorce personas (48,3%) tenían entre 6 y 20 años de experiencia y otras catorce más de 20 años; solo una tenía menos de 5 años (3,5%).

En la Tabla 3 se indican las valoraciones de gravedad de las personas expertas y el número de ellas que consideraron consecuencias potenciales para cada variable.

De las 17 variables incluidas en los registros de enfermería, las omisiones se consideraron con consecuencias muy graves (≥90%) en 10 variables, graves (70-89%) en cuatro y menos graves (<70%) en tres. Las variables más omitidas en los registros prequirúrgicos y postquirúrgicos de enfermería (frecuencia respiratoria, temperatura, glicemia y puntuación EVA) fueron consideradas por los expertos de alta gravedad y con consecuencias, especialmente postquirúrgicas. Las variables de gestión como las altas firmadas y destino del paciente son consideradas de gravedad, pero con mínimas consecuencias. Los resultados en relación con los fármacos muestran valores de omisión en función del total de registros que debían contenerlos según la cirugía, pero al carecer de campos específicos de cumplimentación y ser de anotación libre puede que sean inexactos.

**Tabla 3:** Valoración de la potencial gravedad y consecuencias de los defectos en la cumplimentación de variables de los registros de enfermería

Registros de enfermería - n=491			
Variable	Gravedad %	Consecuencias (n)*	
		IntraQ	PostQ
Alergias del paciente	≥90	26	27
Tensión arterial	≥90	15	26
Frecuencia cardíaca	≥90	16	26
Frecuencia respiratoria	≥90	16	27
Temperatura	70-89	13	28
Glicemia	70-89	20	28
Escala EVA de dolor	<70	7	24
Sonda nasogástrica	<70	11	24
Profilaxis antibiótica	≥90	8	26
Nombre analgésico no opioide	≥90	7	26
Nombre analgésico opioide	≥90	4	27
Nombre Fluidoterapia	≥90	8	27
Test Aldrete	70-89	1	21
Alta de anestesia firmada	≥90	1	12
Alta de Enfermería	≥90	1	11
Destino a la salida del paciente	70-89	1	7
Llamada a la enfermera de hospitalización	<70	1	15

*: número de expertos que consideraron consecuencias potenciales en la seguridad del paciente durante los periodos operatorio (IntraQ) y postoperatorio (PostQ); PreQ: registros prequirúrgicos (a la llegada del paciente); PostQ: registros postquirúrgicos (en la unidad de recuperación post-anestésica); EVA: escala visual analógica.

Las variables más frecuentemente omitidas en los registros de anestesia (relacionadas con la vía aérea, el tipo de anestesia y la ocurrencia o no de incidencias) fueron consideradas graves y con consecuencias. Todas las omisiones en los informes anestésicos tenían consecuencias postoperatorias potenciales según las personas expertas (Tabla 4).

**Tabla 4:** Valoración de la potencial gravedad y consecuencias de los defectos en la cumplimentación de variables de los registros de anestesia

Registros de anestesia (n=491)			
Variable	Gravedad %	Consecuencias (n)*	
		IntraQ	PostQ
Alergias del paciente	≥90	15	27
Tensión arterial	≥90	15	26
Frecuencia cardíaca	70-89	16	26
Vía aérea Mallampati	≥90	13	16
Historia intubación difícil	≥90	19	16
Ventilación facial difícil	≥90	19	22
Distancia tiromentoniana	70-89	15	12
Movilidad cervical	70-89	15	18
Diuresis	70-89	5	25
Peso del paciente	70-89	15	21
Altura del paciente	70-89	6	8
Sonda nasogástrica	70-89	11	24
Fármacos anestésicos	≥90	-	23
Fármaco antitrombótico	<70	-	26
Analgésico no opioide	≥90	-	26
Analgésico opioide	≥90	-	27
Tipo de anestesia	≥90	8	28
Incidencias anestésicas	70-89	7	28
Incidencias hemodinámicas	≥90	10	28
Incidencias respiratorias	≥90	12	28
Incidencias neurológicas	≥90	13	25
Incidencias varias	70-89	8	28

*: número de expertos que consideraron consecuencias potenciales en la seguridad del paciente durante los periodos operatorio (IntraQ) y postoperatorio (PostQ); IntraQ: registros intraquirúrgicos (durante la estancia del paciente en quirófano); PostQ: registros postquirúrgicos (en la unidad de recuperación post-anestésica); -: no aplicable a este registro.

En 20 intervenciones (4,07%) no se había realizado informe quirúrgico, por lo que se analizaron 471 (Tabla 3) que mostraron frecuentes omisiones consideradas graves y con consecuencias. No pudo valorarse el intervalo de tiempo entre el final de la intervención y su cumplimentación.

En las respuestas de *otras consecuencias*, las personas expertas destacaron para las omisiones de registro complicaciones administrativas (n=25), consecuencias legales (n=23) y consecuencias para el manejo de los cuidados enfermeros y gestión del área quirúrgica (n=21) (Tabla 5).

**Tabla 5:** Valoración de la potencial gravedad y consecuencias de los defectos en la cumplimentación de variables del informe quirúrgico

Informe quirúrgico		
Variable	Gravedad	Consecuencias
	%	(n)*
Tipo de anestesia	70-89	25
Tipo de incisión	70-89	16
Cierre de la incisión	70-89	15
Recuento de gasas	70-89	24
Incidencias durante la cirugía	≥90	23
Profilaxis antibiótica	≥90	20
Recogida muestras para anatomía patológica	≥90	15
Recogida de muestras para biopsia	≥90	15
Recogida de muestras para cultivo	≥90	17

*: número de expertos que consideraron consecuencias potenciales en la seguridad del paciente.

A continuación, se presentan los resultados de la encuesta que contestaron los expertos respecto el nivel de gravedad de los errores, las consecuencias y las propuestas de mejoras (Tabla 6). La consistencia interna del cuestionario fue muy alta (alfa de Cronbach=0,995) y el acuerdo de las respuestas del grupo de expertos fue muy alto para todas las variables, con coeficientes de correlación intraclase para valoraciones únicas de 0,880 (0,864-0,895) y para valoraciones promedio de 0,995 (0,995-0,996), ambos con significación estadística (p<0,001).

Los expertos puntuaron muy grave (90%) la omisión de registro de las alergias del paciente, la glicemia, distintos parámetros relacionados con la ventilación mecánica, distintos fármacos, anestésicos e incidencias; profilaxis antibiótica y muestras para cultivo. Las medidas más recomendadas por los expertos para mejorar la cumplimentación de los registros fueron: formaciones telemáticas (41%), formaciones presenciales con metodología de simulación clínica a los equipos profesionales (72%), incorporar alertas de omisión (90%), eliminar los registros en papel (69%) y realizar auditorías periódicas sobre la calidad de los registros (41%) (Tabla 6).

**Tabla 6:** Resultados de la encuesta que los 29 expertos respondieron: dos preguntas para cada variable (P1 y P2) y recomendaciones de mejora

Variable		Nivel de gravedad		Consecuencias en cada fase			
		(P1)		(P2)			
		Media (DE) Rango	%	PreQ	IntraQ	PostQ	Otras
Sección 1.	Proceso Quirúrgico						
1	Alergias del paciente	5 (0,62) R: 4-6	≥90	90	93	93	21
2	Tensión arterial (S/D)	4 (1,50) R: 1-6	70-89	52	93	90	17
3	Frecuencia cardíaca	4 (1,46) R: 1-6	70-89	55	97	90	14
4	Frecuencia respiratoria	3 (1,53) R: 1-6	70-89	55	93	93	14
5	Temperatura	4 (1,71) R: 1-6	70-89	45	83	97	14
6	Glicemia	5 (1,29) R: 2-6	≥90	69	86	97	17
7	Escala EVA de dolor	3 (1,90) R: 1-6	70-89	24	24	83	83
8	Peso del paciente	4 (1,76) R: 1-6	70-89	52	86	72	17
9	Altura del paciente	3 (1,74) R: 1-6	70-89	21	55	28	7
10	Vía aérea Mallampati	5 (1,56) R: 1-6	≥90	45	72	55	10
11	Historia intubación difícil	6 (0,80) R: 3-6	≥90	66	79	55	10
12	Ventilación facial difícil	6 (0,73) R: 4-6	≥90	66	90	76	10
13	Distancia tiromentoniana	5 (1,52) R: 1-6	≥90	52	66	41	14
14	Movilidad cervical	5 (1,55) R: 1-6	≥90	52	76	62	10
15	Diuresis	4 ±1,51) R: 1-6	70-89	17	76	86	14
16	SONDA VESICAL	3 (1,86) R: 1-6	70-89	14	62	86	14
17	Sonda nasogástrica	4 (1,88) R: 1-6	70-89	38	69	83	14
18	BIS	5 (2,04) R: 1-6	≥90	7	62	52	17
19	PVC	4 (1,66) R: 1-6	70-89	31	86	90	14
20	PEEP	5 (1,76) R: 1-6	≥90	14	76	62	14
21	Tipo de anestesia	5 (1,21) R: 1-6	≥90	17	55	86	21
22	Anestesia regional	5 (1,59) R: 1-6	≥90	14	62	90	35
23	Fármacos anestésicos	5 (1,30) R: 1-6	≥90	17	76	93	31
24	Incidencias anestésicas	6 (0,75) R: 3-6	≥90	28	76	97	41
25	Incidencias hemodinámicas	6 (0,76) R: 3-6	≥90	24	79	97	38
26	Incidencias respiratorias	6 (0,81) R: 3-6	≥90	35	76	97	31
27	Incidencias neurológicas	6 (0,77) R: 4-6	≥90	41	79	97	35
28	Incidencias varias	5 (1,06) R: 2-6	≥90	45	79	86	45
29	Test Aldrete	5 (1,49) R: 1-6	≥90	3	7	72	31
30	Test Bromage	5 (1,54) R: 1-6	≥90	3	3	66	31
31	Firmada alta anestesia	4 (2,0) R: 1-6	70-89	3	3	41	86
32	Firmada alta enfermería	4 (2,0) R: 1-6	70-89	3	3	38	83
33	Destino de salida del paciente	4 (2,10) R: 1-6	70-89	3	3	24	86
34	Llamada a hospitalización	4 (1,66) R: 1-6	70-89	3	3	52	69
Sección 2. Farmacología							
35	Anti-trombótico	6 (0,80) R: 4-6	≥90	28	69	90	28
36	Antibiótico profiláctico	5 (1,22) R: 1-6	≥90	24	62	90	41
37	Analgésico no opioide	5 (0,91) R: 4-6	≥90	14	62	93	31
38	Analgésico opioide	5 (0,87) R: 4-6	≥90	28	66	93	28
39	Benzodiacepina	5 (1,50) R: 1-6	≥90	35	69	86	24
40	Fármaco cardiovascular	5 (1,12) R: 2-6	≥90	41	76	90	21
41	Fármaco antiemético	5 (1,34) R: 1-7	≥90	14	62	93	21
42	Fármaco respiratorio	5 (1,33) R: 1-7	≥90	35	69	86	24
43	Fluidoterapia	5 (1,24) R: 2-7	≥90	28	66	93	21
Sección 3. Informe Quirúrgico							
44	Alergias del paciente	6 (0,64) R: 5-7	≥90	35	38	86	48
45	Tipo de incisión	4 (2,0) R: 1-6	70-89	3	3	55	38
46	Cierre de la incisión	4 (1,98) R: 1-6	70-89	3	3	52	35
47	Recuento de gasas	6 (1,43) R: 1-6	≥90	3	14	83	35
48	Incidencias durante la cirugía	6 (1,35) R: 1-6	≥90	3	31	79	35
49	Profilaxis antibiótica	5 (1,62) R: 1-6	≥90	10	31	69	48
50	Recogida muestras para anatomía patológica	5 (1,42) R: 1-6	≥90	3	7	52	72
51	Recogida de muestras para biopsia	5 (1,42) R: 1-6	≥90	3	7	52	72
52	Recogida de muestras para cultivo	5 (1,46) R: 1-6	≥90	3	7	59	66
53	Error de programación: cirugía mayor por cirugía ambulatoria.	4 (2,0) R: 1-6	70-89	10	17	21	59
54	Errores asignación rol: anestesista por cirujano	4 (2,19) R: 1-6	70-89	10	7	14	79
55	Horas de entrada y/o salida	4 (2,11) R: 1-6	70-89	7	10	31	76
Sección 4. Propuestas de Mejora							
P3 ¿Qué medidas podrían ser efectivas para mejorar la calidad en la cumplimentación de los registros quirúrgicos? (puede marcar más de una opción)							
Hacer formaciones telemáticas no sincrónicas a los profesionales implicados.							7 (24%)
Hacer formaciones telemáticas sincrónicas a los profesionales implicados.							5 (17%)
Hacer formaciones con metodología de Simulación Clínica a los profesionales implicados.							21 (72%)
Hacer formaciones con la metodología de Aula Invertida a los profesionales implicados.							9 (31%)
Aplicar algún mecanismo informático para alertar de la cumplimentación deficiente.							26 (90%)
Informatizar todos aquellos registros que aún se cumplimenten en papel.							4 (14%)
Realizar auditorías periódicas.							4 (14%)
Otros.							4 (14%)

P1: ¿Qué nivel de gravedad considera usted que tendría si esta variable no se encuentra marcada/anotada en un registro quirúrgico? (solo puede marcar una opción); P2: ¿En qué fase del proceso quirúrgico podría tener consecuencias/complicaciones si esta variable no está marcada/anotada? (puede marcar más de una opción); Nivel de Gravedad: 1: <50%, 2: 50-69%, 3: 70-79%, 4: 80-89%, 5: 90-99%, 6: 100%, que fueron categorizadas en tres categorías: 1-2: <70% / omisión menos grave, 3-4: 70-89% / omisión grave y 5-6: ≥90% / omisión muy grave.

## DISCUSIÓN

En nuestro concomimiento este es el primer estudio sobre cumplimentación de los registros de proceso en el área quirúrgica que además intenta valorar la importancia de sus deficiencias para la SdP.

Los registros clínicos del proceso quirúrgico son esenciales para que los 394 profesionales que sucesivamente actúan en el proceso conozcan las acciones, tratamientos y respuestas del paciente anteriores a asumir su cuidado. Los registros son herramientas de transferencia entre profesionales esencial para la SdP y la continuidad asistencial^4^^,^^17^. Hay evidencias de la importancia de la calidad de los registros para los resultados clínicos^18^. Para mejorar la calidad de los registros se deben auditar y detectar sus deficiencias, y proporcionar *feedback* sobre ellas a los profesionales^2^^,^^18^^,^^19^.

No se encuentran descripciones de la cumplimentación global de los registros del proceso quirúrgico ni guías de referencia dirigidas a la totalidad de los registros por los distintos profesionales del área quirúrgica que establezcan estándares de cumplimiento. Los documentos existentes se centran en determinadas fases del proceso quirúrgico o ciertos tipos de cirugía, sin considerar que la actuación de los profesionales es secuencial y cada fase está modulada por lo ocurrido en la fase anterior^20^^,^^21^^,^^22^. Esta ausencia de indicadores de calidad generales de los registros de proceso quirúrgico justificó que este estudio obtuviera, a través de expertos, una valoración del peso y consecuencias en la SdP de las deficiencias en los registros del área quirúrgica.

Este estudio unicéntrico demuestra déficits de cumplimentación en registros superiores al 70% en la información sobre aspectos de anestesia, identificación de fármacos administrados, incidencias intercurrentes, datos de la cirugía o de la recogida de muestras biológicas, entre otros. Esto se observó en los registros de enfermería, anestesia y cirugía. La falta de registro de las constantes, alergias y las cinco condiciones de buenas prácticas en la administración de fármacos están declarados por la OMS de gran riesgo para la seguridad del paciente^20^. Los expertos consultados en este estudio coinciden en el alto riesgo de complicaciones pre, intra y postquirúrgicas que pueden surgir cuando el profesional que atiende al paciente no dispone de ellos porque en la anterior fase del proceso no se han anotado. Cuando se omiten estos datos los profesionales están actuando fuera del mandato, elevando el riesgo de producir efectos adversos. En caso de complicaciones, la falta de constancia en el registro de alguna acción o dato previo necesario podría tener graves consecuencias médico-legales para profesionales e institución.

Los registros que más variables contienen (los de enfermería y anestesia) eran en papel, lo que provoca ilegibilidad. Carecemos de información publicada que permita comparar nuestra incidencia de ilegibilidad.

La ausencia de campos específicos para anotar la administración de fármacos ha dificultado valorar la calidad de su cumplimentación, de suma importancia para la SdP. Sin embargo, también el registro quirúrgico electrónico presentó omisiones frecuentes y graves, lo que muestra que actuar solo sobre el soporte de un registro no evita las omisiones.

La ausencia de antecedentes en este campo dificulta establecer qué variables deben ser cumplimentadas en cada fase del proceso, al igual que el nivel tolerable de omisión. Aun considerando que fijar un nivel de corte es arbitrario, en las respuestas de los expertos al cuestionario de este estudio hemos considerado a partir de un 70% de omisión como un error grave o muy grave, basándonos en una publicación de 2014 sobre informes de enfermería^19^, la única hallada.

Aparte de identificar deficiencias graves en la cumplimentación de los registros de nuestro centro y poder priorizar las dianas de una estrategia de mejora institucional, hemos evidenciado mejoras importantes en su diseño. Como sucede en la mayoría de las instituciones, los registros del proceso quirúrgico se han diseñado en función de los roles profesionales y no de la disponibilidad de información compartida, y esto hace que, entre ellos, haya mucha repetición innecesaria de las variables a registrar. Pero, además, se repite información previa de la que ya se dispone en la historia clínica, disponible para el personal a la llegada del paciente al quirófano. Un ejemplo son los parámetros de valoración de la vía aérea que se evalúan en la visita preanestésica previa y que se omiten en la mitad de los informes; bastaría con indicar en el registro de quirófano si la intubación se prevé difícil. Otro ejemplo es la repetición de datos importantes incluidos la LVSQ que, dado su diseño universal y su obligado cumplimiento^10^, puede hacer que el profesional vea innecesario repetirlos en otro registro. Es el caso del recuento de gasas o de la recogida de muestras para laboratorios, datos que figuran como omitidos cuando estaban disponibles en la historia. Sin embargo, hay datos con alto porcentaje de omisión, como comunicar si hubo o no incidencias intercurrentes, que pueden ser cruciales para los cuidados postoperatorios del paciente y para anestesias y cirugías posteriores, y que solo el personal anterior puede informar. La falta de coordinación y coherencia entre los diferentes registros de cada fase hacen difícil valorar la importancia de cada omisión detectada en los resultados sobre el paciente.

En nuestra opinión, los registros de los distintos profesionales del área quirúrgica deberían incluirse en un único informe electrónico para que cada profesional pudiera consultar el del resto. Además, se debería registrar la identificación del profesional que lo cumplimenta, y los parámetros que permanecen estables en todo el proceso (como antecedentes, vía aérea o profilaxis antibiótica) se trasladarían automáticamente entre los informes del registro. Los parámetros que varían a lo largo del proceso (como constantes vitales, procedimientos realizados e incidencias) deberían registrarse en cada fase, pero serían conocidos por los subsiguientes profesionales cuando accedieran a realizar sus informes. La administración de fármacos debería tener casillas de obligada cumplimentación (nombre, dosis, vía).

Este estudio valoró la frecuencia en que una variable no se registra pero también la gravedad de que no conste. Hay variables con alta frecuencia de omisiones que no revisten gravedad (existencia de sonda nasogástrica, altura del paciente) mientras que la omisión de otras (alergias) puede tener consecuencias graves. La temperatura y la glicemia, frecuentemente omitidas en los registros revisados, figuran en guías y recomendaciones basadas en la evidencia^14^^,^^23^, y son variables que pueden cambiar a través de las distintas fases del proceso quirúrgico. Estos conceptos son importantes para establecer la priorización dentro de la estrategia de mejora basada en indicadores de calidad de los registros.

La coincidencia de criterio de los expertos en referencia a la gravedad y consecuencias de las omisiones e ilegibilidad de las variables y sobre las propuestas de mejora, fue altamente consistente. Esta consistencia y la experiencia y representatividad del grupo de expertos, contrarrestan en parte la limitación de su número y permite establecer prioridades de la estrategia de mejora institucional, que en nuestra institución se centrará en la consideración conjunta de frecuencia de omisión, gravedad y consecuencias potenciales.

Los expertos coinciden en apoyar la formación presencial de los equipos y tomar acciones sobre el diseño de los registros, que deben ser electrónicos. Sugerimos también la cumplimentación electrónica de la LVSQ y que esté ligada a este registro global. Los registros clínicos en soporte electrónico ofrecen ventajas indudables sobre los realizados en papel, que frecuentemente presentan dificultades de legibilidad^24^^,^^25^. No obstante, deben revisarse sus diseños para que cumplan su objetivo de transferencia y aumentar su aceptabilidad entre profesionales que, con frecuencia, consideran que sobrecargan su trabajo^26^^,^^27^. Por ello, creemos que los nuevos registros informatizados deberían obligar a contestar cada variable, al menos las consideradas como de alto impacto, mediante imposibilidad de seguir con el informe o con alertas de omisión.

Hay que considerar varias limitaciones en relación a este estudio. Las variables clínicas de los registros se evaluaron por la ausencia o ilegibilidad de datos, sin relacionarlos con su repercusión sobre los resultados clínicos, que se aproximó mediante la opinión de una muestra de expertos que fue solo del 38,6% de los previstos, pero representativa de los profesionales que intervienen. No se incluyó la información que registra la LVSQ que, aunque no sale del quirófano, el profesional ya ha registrado en ella datos que se repiten en los registros clínicos, lo que podría inducir a los profesionales a omitirlos. La validez externa de los resultados de este estudio es limitada ya que corresponden a una única institución y sus modelos de registro. Sin embargo, el proceso de análisis se podría replicar en otros centros que presenten el mismo problema de fraccionamiento y falta de coordinación de la información entre los distintos registros del proceso en el área quirúrgica, aunque sus registros sean diferentes.

Las deficiencias en la cumplimentación de los registros del proceso quirúrgico han sido frecuentes y muchas de ellas con potenciales consecuencias para el paciente. La falta de indicadores estandarizados para el control y seguimiento de los registros impide poder hacer un buen control de su calidad. No obstante, los indicadores de calidad del registro de medicación están bien definidos y fueron muy deficientes en nuestros registros.

En conclusión, el panel de expertos ha considerado que los defectos de cumplimentación de los registros quirúrgicos de enfermería, anestesia y cirugía respecto a datos clínicos, fármacos administrados, incidencias recurrentes, datos de cirugía o recogida de muestras biológicas, han supuesto un gran riesgo para la seguridad de los pacientes atendidos durante el proceso quirúrgico. Este estudio ha evidenciado la presencia de un alto riesgo de padecer complicaciones quirúrgicas que pueden derivar en graves consecuencias médico-legales tanto para profesionales como para la institución. Las personas expertas también han considerado la formación mediante simulación clínica, entre otras, como una herramienta eficaz para mejorar la calidad de la cumplimentación de los registros.

## References

[1] Institute of Medicine (US) Committee on Quality of Health Care in America (2000). To err is human: Building a safer health system.

[2] Franco Vega MC Aiss MA, George M Day L, Mbadugha A, Owens K (2024). Enhancing implementation of the I-PASS handoff tool using a provider handoff task force at a comprehensive cancer center. Jt Comm J Qual Patient Saf.

[3] Ballester Roca M (2020). ¿Estamos abordando el problema de la seguridad del paciente en los hospitales desde la perspectiva correcta?. Folia Humanistica.

[4] Bernia JA, Roure C, Broto A (2013). Prevención de errores de medicación en paciente quirúrgico. Boletín de prevención de errores de medicación de Cataluña.

[5] Peláez R, Aguilar JL, Segura C, Fernández S, Mendiola MA, Forner JC (2009). Experiencia de un equipo interdisciplinar de Anestesiología y Enfermería en un circuito de anestesia fuera de quirófano. Rev Esp Anestesiol Reanim.

[6] Saarinen I, Malmivaara A, Miikki R, Kaipia A (2018). Systematic review of hospital-wide complication registries. BJS Open.

[7] Medical Protection Medical records: uses.

[8] Scarpis E, Brunelli L, Tricarico P, Poletto M, Panzera A, Londero C (2021). How to assure the quality of clinical records? A 7-year experience in a large academic hospital. Plos One.

[9] National Health Service Health Education England. Record keeping - the consequences of recording/documenting patient information incorrectly.

[10] Haynes AB, Weiser TG, Berry WR, Lipsitz SR, Breizat AH, Dellinger EP, Safe Surgery Saves Lives Study Group (2009). A surgical safety checklist to reduce morbidity and mortality in a global population. N Engl J Med.

[11] Evans SM, Scott IA, Johnson NP, Cameron PA, McNeil JJ (2011). Development of clinical-quality registries in Australia: the way forward. Med J Aust.

[12] Lee P, Chin K, Liew D, Stub D, Brennan AL, Lefkovits J (2019). Economic evaluation of clinical quality registries: A systematic review. BMJ Open.

[13] Gobierno de España. Ministerio de Sanidad Seguridad del paciente. Programa de seguridad en el bloque quirúrgico.

[14] Servei Català de la Salut CatSalut PREVINQCAT.

[15] Khurshid Z, De Brún A, Martin J, McAuliffe E (2021). A systematic review and narrative synthesis: Determinants of the effectiveness and sustainability of measurement-focused quality improvement trainings. J Contin Educ Health Prof.

[16] Fiallos S (2024). Simulación clínica en la formación de profesionales de la salud: explorando beneficios y desafíos. Revista Científica de Salud y Desarrollo Humano.

[17] Stey AM, Russell MM, Ko CY, Sacks GD, Dawes AJ, Gibbons MM (2015). Clinical registries and quality measurement in surgery: a systematic review. Surgery.

[18] Van der Veer SN, de Keizer NF, Ravelli ACJ, Tenkink S, Jager KJ (2010). Improving the quality of care. A systematic review of how medical registries provide information feedback to health care providers. Int J Med Inform.

[19] Hoque DME, Kumari V, Hoque M, Ruseckaite R, Romero L, Evans SM (2017). Impact of clinical registries on quality of patient care and clinical outcomes: A systematic review. Plos One.

[20] Gobierno de España. Ministerio de Sanidad, Servicios Sociales e Igualdad Estrategia de Seguridad del Paciente del Sistema Nacional de Salud Período 2015-2020.

[21] Gobierno de España. Ministerio de Sanidad, Política Social e Igualdad (2010). Guía de Práctica Clínica para la Seguridad del Paciente Quirúrgico.

[22] Ortega Vargas C, Quintero Barrios MM, Suárez Vázquez MG, Solís Pérez MT, Jiménez y Villegas MC, Zárate Grajales RA (2014). Manual de evaluación de la calidad del servicio de enfermería. Estrategias para su aplicación.

[23] National Institute for Health and Care Excellence NICE (2008). Clinical guideline [CG65]. Hypothermia: prevention and management in adults having surgery.

[24] Kandaswamy S, Hettinger AZ, Hoffman DJ, Ratwani RM, Marquard J (2020). Communication through the electronic health record: frequency and implications of free text orders. JAMIA Open.

[25] Gobierno de España. Agencia de Calidad del Sistema Nacional de Salud (2008). Conjunto Mínimo de Datos en los Informes Clinicos. Historia Clínica Digital en el Sistema Nacional de Salud.

[26] Wright A, McGlinchey EA, Poon EG, Jenter CA, Bates DW, Simon SR (2009). Ability to generate patient registries among practices with and without electronic health records. J Med Internet Res.

[27] Rosenbaum BP, Lorenz RR, Luther RB, Knowles-Ward L, Kelly DL, Weil RJ (2014). Improving and measuring inpatient documentation of medical care within the MS-DRG system: Education, monitoring, and normalized case mix index. Perspect Health Inf Manag.

